# Supplementary material for: FERN – a Java framework for stochastic simulation and evaluation of reaction networks
Source: BMC Bioinformatics. 2008 Aug 29;9:356. doi: 10.1186/1471-2105-9-356 (PMC2553347; doi:10.1186/1471-2105-9-356)
Supplement: Additional file 1 — FERN distribution, Version 1.3. This archive contains the FERN source code and binaries as well as documentation and example models in FernML and SBML. [file 1471-2105-9-356-S1.zip › fern/doc/javadoc/fern/simulation/algorithm/TauLeapingRelativeBoundSimulator.html]

TauLeapingRelativeBoundSimulator


---


|  |  |  |
| --- | --- | --- |
| **Overview** \| **Package** \| **Class** \| **Use** \| **Tree** \| **Deprecated** \| **Index** \| **Help** | |  |
| **PREV CLASS**   **NEXT CLASS** | **FRAMES**    **NO FRAMES**     **All Classes** |  |
| SUMMARY: NESTED \| FIELD \| CONSTR \| METHOD | DETAIL: FIELD \| CONSTR \| METHOD |  |


---


## fern.simulation.algorithm Class TauLeapingRelativeBoundSimulator

```
java.lang.Object
  fern.simulation.Simulator
      fern.simulation.algorithm.GillespieEnhanced
          fern.simulation.algorithm.AbstractBaseTauLeaping
              fern.simulation.algorithm.AbstractTauLeapingPropensityBoundSimulator
                  fern.simulation.algorithm.TauLeapingRelativeBoundSimulator
```

---

``` public class TauLeapingRelativeBoundSimulator extends AbstractTauLeapingPropensityBoundSimulator ```

There are some possibilities to bind the expected change of the propensities by a
value epsilon in order to fulfill the leap condition. Here the expected change is bound
to individual propensities.

Daniel T. Gillespie, Approximate accelerated stochastic simulation
of chemically reacting systems, Journal of chemical physics vol 115, nr 4 (2001); Cao et al., Efficient
step size selection for the tau-leaping simulation method, Journal of chemical physics 124, 044109 (2006)

**Author:**
:   Florian Erhard

---

**Nested Class Summary**

**Nested classes/interfaces inherited from class fern.simulation.Simulator**

`Simulator.FireType`


**Field Summary**

**Fields inherited from class fern.simulation.algorithm.AbstractTauLeapingPropensityBoundSimulator**

`f, mu, sigma`

**Fields inherited from class fern.simulation.algorithm.AbstractBaseTauLeaping**

`productHistos, reactantHistos, verbose`

**Fields inherited from class fern.simulation.algorithm.GillespieEnhanced**

`a_sum`

**Fields inherited from class fern.simulation.Simulator**

`a, stochastics, t`


**Constructor Summary**

`TauLeapingRelativeBoundSimulator(Network net)`


| **Method Summary** | |
| --- | --- |
| `String` | `getName()`             Returns the name of this simulator |
| `protected  double` | `getTop(int j)` |

**Methods inherited from class fern.simulation.algorithm.AbstractTauLeapingPropensityBoundSimulator**

`chooseTauNonCriticals`

**Methods inherited from class fern.simulation.algorithm.AbstractBaseTauLeaping**

`chooseTauCriticals, getEpsilon, getLangevinThreshold, getNCritical, getNumSimpleCalls, getUseSimpleFactor, getV, isVerbose, performStep, setEpsilon, setLangevinThreshold, setNCritical, setNumSimpleCalls, setUseSimpleFactor, setVerbose`

**Methods inherited from class fern.simulation.algorithm.GillespieEnhanced**

`directMCTau, initialize, isEfficientlyAdaptSum, reinitialize, setAmount, setEfficientlyAdaptSum, setVolume`

**Methods inherited from class fern.simulation.Simulator**

`addObserver, fireReaction, fireReaction, getAmount, getAmountManager, getNet, getNextThetaEvent, getPropensity, getPropensityCalculator, getTime, getVolume, isInterpolateTheta, registerNewTheta, setInterpolateTheta, start, start, thetaEvent`

**Methods inherited from class java.lang.Object**

`clone, equals, finalize, getClass, hashCode, notify, notifyAll, toString, wait, wait, wait`

**Constructor Detail**

### TauLeapingRelativeBoundSimulator

```
public TauLeapingRelativeBoundSimulator(Network net)
```


**Method Detail**

### getTop

```
protected double getTop(int j)
```

:   **Specified by:**: `getTop` in class `AbstractTauLeapingPropensityBoundSimulator`

---


### getName

```
public String getName()
```

:   **Description copied from class: `AbstractBaseTauLeaping`**
:   Returns the name of this simulator

    :   **Overrides:**: `getName` in class `AbstractBaseTauLeaping`
    :   **Returns:**: simulator name


---


|  |  |  |
| --- | --- | --- |
| **Overview** \| **Package** \| **Class** \| **Use** \| **Tree** \| **Deprecated** \| **Index** \| **Help** | |  |
| **PREV CLASS**   **NEXT CLASS** | **FRAMES**    **NO FRAMES**     **All Classes** |  |
| SUMMARY: NESTED \| FIELD \| CONSTR \| METHOD | DETAIL: FIELD \| CONSTR \| METHOD |  |


---
